# Organizing Virtual Care, Digital Services Replacing Hospital In-Care and Outpatient Care

**DOI:** 10.1016/j.mcpdig.2024.06.007

**Published:** 2024-07-08

**Authors:** Wim van Harten, Carine Doggen, Laura Kooij

**Affiliations:** a Department of Health Technology and Services Research, University of Twente, Enschede, The Netherlands; b Science Department, Rijnstate Hospital, Arnhem, The Netherlands; c Netherlands E-health Living Laboratory, Leiden University, Leiden, The Netherlands

## Abstract

Hospital-based digital care and virtual care are becoming increasingly common and their reach and scope are expanding in terms of patient groups and technological sophistication. The objective of this viewpoint is to provide guidance on design and factors that can be decisive for the organization of virtual care from a hospital’s perspective. Relevant aspects to be taken into account are as follows: characteristics of the technology, in a broader sense, the nature and intensity of provider involvement and supervision, the degree of self-management by the patient and his environment, the relation and cooperation mechanisms with other providers as home care, general practitioner ’s and other specialist care, the matter of (economies of) scale and finally the uniformity of processes over geographic regions and providers. We provide suggestions for further research and future policy related to these aspects.

Hospital-based digital care and virtual care are becoming increasingly common and their reach and scope are expanding in patient groups and technological sophistication. In the United States, this concerns various digital health services and digital technology implementation in remote monitoring and medical and surgical home programs.^1^, ^2^, ^3^ In the United Kingdom, hospitals are required to implement at least 1 type of virtual ward.^4^ By virtual care we mean care commonly linked to the physical location of the hospital, that is completely or in part provided remotely and with digital technology; also described as digital care. The patient plays a role by taking regular measurements at home or completing questionnaires in an application. A (wireless) sensor or wearable can be used that continuously takes measurements for staff or patients to act on. If necessary, video or telephone contact takes place. Often there is a link with hospital at home services, involving hospital staff like specialized nurses, and physical therapists, or in a hybrid form involving home care services.^5^^,^^6^ There is no accepted uniform definition of digital care or virtual care,^7^ in this article we use virtual care.

In many Dutch hospitals virtual care centers are expanding their services, especially after the COVID-19 crisis. They monitor patient’s vital signs and a range of symptoms and guide patients remotely, sometimes after early discharge. Examples are early discharge after hospitalization for COPD, pancreatitis or infectious diseases or same day discharge after bariatric surgery. Patients receive intensive guidance for several days through a combination of monitoring vital signs with a (wireless) device, education, and instruction for the patient or his informal caregivers with applications. In addition, digital or telephone contact with a nurse or other health care professionals is foreseen. As a result, patients can go home earlier and hospital capacity is used more efficiently. Although evaluation of patient experiences in our hospital shows that patients are very satisfied with this care, reviews are not conclusive.^8^

Virtual care is also used for patients with chronic conditions to decrease the frequency of hospital visits and improve self-management and gradually more diagnostic groups, such as diabetes, COPD, or chronic heart failure, are included. The programs use a mix of approaches to support self-management and use uploading of self-registered-based data or questionnaire or survey-based data. In addition, automated wearable or device-based monitoring can be used, enabling virtual care staff to assess the need to adapt the treatment or to seek contact with the patient on indication.

An important question is how to design and organize virtual care. In literature we cannot find explicit descriptions of the organizational setting nor the factors determining service design, such as details on technical platforms, degree of integration with the electronic medical record (EMR), staffing and integration in or coordination with the home care or general practice services. Important questions are the degree of their involvement in terms of cooperation or make-or-buy decisions.

Because there is a wide variation in types of virtual care, treatments and diagnoses involved, this influences design and organizational aspects. It makes a difference whether, in the case of heart failure, an intravascular pressure sensor sends data to a central location with a specialist expertise, or whether the patient completes a questionnaire or self-measured value in the application once a month.

Policymakers often have high expectations about possible savings, however, we found little solid research into efficiency claims. In July 2022, we published a budget impact analysis in BMJ Open^9^ about virtual care to shorten hospital admissions using device-based remote monitoring (measuring equipment that wirelessly sends patient data) based on one hospital case and projected on different scenarios. In the Dutch setting, it depends very much on the scale of activities whether a positive business case can be achieved from a hospital perspective. The costs of technology, personnel, and organization should not be underestimated. It is not yet clear whether staff can reliably serve very different patient groups in the post-acute phase. If nursing deployment cannot be reduced by reducing shifts, for example by limiting the number of contacts or by using algorithms or artificial intelligence (AI), economies of scale is necessary to achieve savings. Joint investment in technology, centralizing care in one physical location or a larger scale virtual organization can then be solutions.

In a systematic literature review on the effects of digital care on health care capacity use in a range of diseases, we found mixed but mostly moderate effects in reducing hospital admissions, length of stay, and outpatient use.^10^ Most studies do not report on actual technological settings and service design, nor on the way of integration in normal hospital operations. Commonly random controlled trials do not report whether the digital service was continued or covered by insurance after the trial. And if the capacity gains per diagnostic group, trial or pilot setting are reported, it is seldom in numbers that lead to actual cost reduction for the health system. It is therefore relevant to list the relevant aspects that are decisive in organizing virtual care from an organizational and economic perspective.

The objective of this article is to provide guidance on design and organizational factors relevant to the organization of virtual care, from a hospital’s perspective. This is based on years of research in digital care, our experiences in one of the earliest virtual care centers in The Netherlands and in a consortium of large teaching hospitals^11^ and on research cooperation with a (Technical) University.

## Relevant Aspects

Depending on the targeted patient population, type of treatment, and the complexity of care, various aspects are relevant for an efficient organizational design of virtual care.

(1) The nature of the technology. This can differ considerably: complex monitoring can start immediately after accelerated discharge, for example after surgery and vital values (such as heart rate) are continuously monitored with a sensor. Less acute patients can be equipped with implantable devices and wearable devices and chronic patients are provided with low-technological applications that only need uploading of self-measured-based data or survey-based data. The technical infrastructure and level of support vary accordingly. Is internet access at home conditional? Is integration with the EMR needed to avoid extra clicks and to enable an integrated approach using up-to-date EMR data for health care professionals? In terms of connectivity, do other providers derive information or add actual information? Does device-based remote monitoring demand specific platform technology or is connectivity provided through applications from commercial providers? How is safety guaranteed in this complex environment, in terms of data protection and avoiding risk of cyberattacks?
(2) The nature of the supervision can differ from high-frequency monitoring of vital signs with (virtual) contact immediately after discharge, to low-frequency for outpatients, especially long-term patients, in the self-management phase. How frequent, for how long, and by which professional are important questions. The physician responsible for the patient must rely on the monitoring system and on the virtual care center staff with sufficient expertise in the disease in question. Remote home monitoring with the support of AI and algorithms may ultimately be carried out by less specialized staff provided evidence-based algorithms or (simpler) sets of rules will become available in combination with adequate backup services.
(3) The degree of self-management. This is limited in the case of early hospital discharge, compared to the chronic outpatient setting and depends on the type of disease. The use of devices to measure and of an application to enter a limited number of values (such as weight and blood pressure) is usually combined with instruction or educational material that supports self-management. The feasibility of virtual care therefore also depends on the (digital) health literacy and digital access and capacities of patients and informal caregivers.
(4) The position and availability of general practitioners (GPs) in the health system can differ very much; are GPs active, do they have a gatekeeper type of role or can patients choose to involve a specialist directly? The relationship between home care and the GP requires agreement on pathway design, shared care services, responsibilities, and roles of the various providers. In some systems the GP or homecare agencies are active and keep taking care of other chronic conditions and problems parallel to the hospital-based virtual care services. The virtual care patient resides in the domain of the general practitioner while being digitally supervised by the hospital or specialist physician for a specific health issue. When freestanding specialist practices or competing hospital providers are also providing virtual care there is a question of regional alignment. Both about GPs, home care and other providers, access to and connectivity in the platforms have to be arranged without compromising the cybersecurity. In view of those different interactions, regional collaboration and aligning with other providers on care pathways, roles and responsibilities is of great importance.
(5) Economics and economies of scale. Hospital financing can differ between fee-for-service and fixed budget models, the degree to which lost in-patients’ days can be recovered by serving additional patients and the existence of adequate reimbursement for virtual care. The GPs might protest if their financing model does not accommodate shifting patients from the hospital. In some health systems, all out-of-hospital services should be performed by home care agencies or GPs through their reimbursement arrangements; in other systems, hospitals can be faced with make-or-buy decisions.

The numbers of patients involved per diagnostic group, -pathway and technology strongly influence the economic feasibility of virtual care programs. The caseload per professional can differ about activities needed and the investments are more easily depreciated covering larger populations. Reviews mostly report on the reduction of capacity use per diagnostic group^10^^,^^12^ and seldom report on actual savings for the health system as a whole. Reviews on cost effectiveness find mixed results and even a positive incremental cost effectiveness ratio often means incremental costs for the system as a whole^13^.

(6) Finally, there is the question of how uniform the detailed process steps in pathways can and need to be organized throughout hospital catchment areas or larger regions covering different hospitals. A national or syst mwide monitoring approach for the follow-up of patients with a fracture or diabetes mellitus requires a uniform remote monitoring process. A regional virtual care center cooperating with several GPs needs uniformity on that scale through agreements between hospitals, general practitioners, and other primary care providers.

## Case Examples of Organization of Virtual Care in Practice

### Smaller Numbers of Patients, Early Discharge, and High-Frequency Monitoring

As long as there is little or no self-management, high-technology monitoring and only short-term high-frequency contact with a health care professional, for example after early discharge from the hospital after surgery or acute pancreatitis, the conclusion from our budget impact study is relevant. Economies of scale in cooperation with other providers through shared services should be sought, such as in the deployment of staff and investments in technology.

As specialized wards and staff were involved in inpatient care, the question is how to guarantee expertise for the specific conditions and whether AI can play a role in generating notifications. In collaboration with other providers around these specialized care pathways, uniformity of the process, of organizational design and technological infrastructure are major issues.

#### Larger Numbers of Patients, Regional Collaboration, and Self-Management Approach

Less frequent monitoring and an approach that is strongly based on self-management involves larger numbers of patients, for which good embedding of the service in the region is important. The benefit is not so much in inpatient capacity but mainly in outpatient clinic capacity and staffing that can be used differently, for example for COPD, asthma patients or type 1 and 2 diabetes care.

This is quickly profitable for a large hospital and smaller hospitals by exploring collaboration with other providers. Uniformity is important here, but rather on the scale of the partnership. Regarding costs and servicing by commercial technology partners, each region should not invent its own wheel for each care path to enable technology standardization.

#### Low Numbers per Hospital, Complex Monitoring Technology, and Chronic Care

If the number of patients per hospital is low, the monitoring technology specific and complex, and personalized advice is needed, these services should be organized for larger populations. Examples are service desks for implantable heart rate monitoring and virtual care for complex patients with diabetes using a bi-hormonal closed loop system.^14^^.^ Monitoring can be centralized in combination with tailored support for connected hospitals and treatment teams. The optimal balance between centralized services and local backup and support is yet to be established.

In Table 1 we provide the relevant factors and related types of care.

**Table 1:** Relevant Aspects for Designing and Organizing Virtual Care

Relevant aspects		
Technology	Type	High technology, eg, wearable sensors especially for complex patient populations, such as patients with pancreatitis. Low technology, eg, applications especially for (chronic) outpatients such as patients with diabetes.
	Infrastructure	Integration with the electronic medical records. Connectivity with systems from other providers.
	Level of technical support	For platform or applications is dependent on technology type and infrastructure (implanted rhythm detection versus COPD survey scores).
Supervision	Frequency: monitoring and contact	High frequency eg, continuous vital signs monitoring especially for complex patient population (early discharge), such as infectious diseases. Low-frequency, eg, self-management especially for (chronic) outpatients, such as patients with chronic kidney disease.
	Type of staff	Physicians. Virtual care staff: specialized nurses. Less specialized (virtual care) staff—general helpdesk like solutions, supported by evidence-based algorithms.
Self-management	Degree	Limited for clinical (complex) patient populations eg, early hospital discharge for urosepsis treatment. High for chronic outpatients: vital parameter monitoring is combined with educational material in COPD; centrally supported self-management and phial replacement in diabetes type 1.
	(Digital) health literacy	Degree affects accessibility and feasibility of virtual care (reading instructions, answering surveys, and acting on alerts).
	Family and relatives	Degree of involvement and dependence of informal caregivers.
Relationship GP and home care	Type	Strength and domain-specific role of GP and home care services; especially relevant in dealing with comorbidities, parallel medical issues, and in immediate situations.
	Organization	Requires agreement on pathway design, shared care services, and provider roles.
Economics and economies of scale	Hospital finance	Existence of adequate reimbursement and adequate internal costing model.
	Role of GP	Financing model for GP and home services in transfer of patients
	Feasibility	Dependent on technology costs, number of patients involved per diagnostic group,—pathway and per intervention.
Uniformity of remote patient monitoring processes	Area	Hospital catchment area, hospital system or nationwide. Technology (platform) and pathway dependent uniform remote monitoring process. Cooperation with other regional providers through (service level) agreements and efforts to reach consensus.

Abbreviations: COPD, chronic obstructive pulmonary disease; GP, general practitioner

## Further Research and Future Policy

Technologies are constantly evolving and new technical features are brought on the market, whereas the underlying evidence about effectiveness is usually lagging. The physical boundaries of the hospital are becoming blurred as virtual care can start directly after an outpatient or emergency department contact, or as a continuation of monitoring as a service for any patient within the hospital. Cooperative models with other providers are also likely to emerge. It is therefore wise not to take too firm positions on the optimal organizational design for virtual care yet.

Evidence-building around the previously mentioned aspects is of great importance. Options for standardizing processes and technology, AI support, and the design of collaboration with health care providers in the region or with other hospitals need to be explored. Collaboration with general practitioners and home care agencies is of great importance in view of seamless care and addressing comorbidities. Socio-dynamic aspects related to cooperation are usually underestimated and not well explored in virtual care. Nurses might fear changes in their work. There can be resistance with GPs to having to adhere to an infrastructure chosen by hospitals without previous participation in decision-making. In most regions, this collaboration and the technological infrastructure are still in its infancy and the conversation about it has only just started.

A very important, but (still) unanswered question is whether virtual access to care and self-measurements can generate latent or even unnecessary care needs, especially when using permanent monitoring through wearables or when digital access is easier than physical access. A recent paper on the impact of telemedicine on Medicare spending and care utilization in the Covid years showed a 2.2% increase in outpatient and telemedicine contacts and a 1.6% cost increase over 2019-2022.^15^ It is relevant to remain critical on the appropriateness of virtual care.

We feel that all these aspects must be taken into account when designing and organizing virtual care to actually benefit from the promises the technology holds in terms of patient-centered, effective, and efficient care.

## Potential Competing Interests

The authors have no conflicts of interest to disclose.
