# Supplementary material for: Organizing Virtual Care, Digital Services Replacing Hospital In-Care and Outpatient Care
Source: Mayo Clin Proc Digit Health. 2024 Jul 8;2(3):405–10. doi: 10.1016/j.mcpdig.2024.06.007 (PMC11975975; doi:10.1016/j.mcpdig.2024.06.007)
Supplement: Supplementary Material [file mmc1.docx]

**Organizing Virtual Care, Digital Services Replacing Hospital In- and Outpatient Care**

Authors: Wim van Harten^1,2,^, Carine Doggen^1,2^, Laura Kooij^2,3^

1. Dept of Health Technology and Services Research, University of Twente, Enschede The Netherlands
2. Rijnstate Hospital, Arnhem The Netherlands
3. Netherlands E-health Living Lab, Leiden University, Leiden, The Netherlands

**CRediT author statement**

**Herewith I confirm that all authors have been equally involved in conceptualisation, writing, reviewing and editing of this paper.**

**All authors have also been involved in underlying research.**

**Prof. Wim H van Harten MD., PhD.**

**Enschede April 2024**
